# Organ Involvement and Constitutional Symptoms in Idiopathic Hypereosinophilic Syndrome: A 72-Patient Single-Centre Cohort

**DOI:** 10.3390/jcm15145569

**Published:** 2026-07-16

**Authors:** Piotr Łacwik, Izabela Kupryś-Lipińska, Aleksandra Kucharczyk, Antonio Rosato, Magdalena Adamczewska, Piotr Damiański, Dominika Ochab, Anna Mościcka, Piotr Lichota, Maciej Kupczyk, Cezary Pałczyński, Piotr Kuna

**Affiliations:** 1 Collegium Medicum, Jan Kochanowski University, 25-317 Kielce, Poland; 2 Clinical Division of Lung Diseases and Allergology, Holy Cross Centre for Lung Disease, 26-060 Chęciny, Poland; 3 Clinical Department of Internal Medicine, Asthma and Allergy, Medical University of Lodz, 90-419 Lodz, Polandpiotr.kuna@umed.lodz.pl (P.K.); 4 Department of Internal Medicine, Pneumonology, Allergology and Clinical Immunology, Military Institute of Medicine, 04-141 Warsaw, Poland; 5 Department of Surgery, Oncology and Gastroenterology (DiSCOG), University of Padua, 35128 Padua, Italy; 6 Veneto Institute of Oncology (IOV-IRCCS), 35128 Padua, Italy; 7 Clinic of Pneumonology, Medical University of Lodz, 90-419 Lodz, Poland; 8 Clinical Department of Otorhinolaryngology, Integrated Provincial Hospital, 25-736 Kielce, Poland

**Keywords:** hypereosinophilic syndrome, idiopathic hypereosinophilic syndrome, eosinophilia, organ involvement, fatigue, constitutional symptoms, asthma, eosinophilic myocarditis

## Abstract

**Background:** Idiopathic hypereosinophilic syndrome (iHES) is a rare disorder of persistent hypereosinophilia with secondary organ damage, but the full spectrum of organ involvement and the constitutional-symptom burden under systematic ascertainment remain incompletely characterised. **Objectives:** To describe the clinical phenotype of iHES in a large single-centre cohort, with attention to symptoms typically under-reported in observational series. **Methods:** We followed 72 consecutive patients with iHES at a tertiary referral centre between January 2017 and May 2025. Defined HES variants were excluded by FIP1L1-PDGFRA RT-PCR and aberrant T-cell phenotyping on flow cytometry; secondary causes were excluded clinically. Each potential organ manifestation was systematically assessed at every visit using a structured clinician-administered checklist. Wilson 95% confidence intervals (CIs) are reported for all proportions. **Results:** Median age at diagnosis was 45 years (interquartile range [IQR] 31–58); 83% were female. The median number of involved organ systems was 3 (IQR 2–4, range 1–6). Pulmonary (91.7%, 95% CI 83.0–96.1), sinonasal (75.0%, 95% CI 63.9–83.6), musculoskeletal (72.2%, 95% CI 61.0–81.2) and gastrointestinal (66.7%, 95% CI 55.2–76.5) involvement predominated. Constitutional symptoms—principally fatigue—were recorded in 97% of patients (95% CI 90.4–99.2) and tracked clinically with disease activity. Cardiac involvement was infrequent (8.3%, 95% CI 3.9–17.0) but uniformly severe. **Conclusions:** Under systematic symptom probing, constitutional features and pulmonary/sinonasal involvement were recorded more frequently in this single-centre idiopathic HES cohort than in earlier series that pooled HES variants. However, selection bias from a pulmonology and severe-asthma referral pattern, together with differences in symptom ascertainment, likely contributes to these estimates and limits their generalisability to the broader iHES population. Routine echocardiographic screening and structured assessment of fatigue and cognitive symptoms should be incorporated into iHES monitoring.

## 1. Introduction

Idiopathic hypereosinophilic syndrome (iHES) is defined by sustained eosinophilia (≥1.5 × 10^9^/L for ≥1 month), resulting in organ damage in the absence of an identifiable secondary cause [1,2]. The diagnosis is largely one of exclusion of other defined hypereosinophilic disorders, including myeloproliferative and lymphocytic variants [3,4]. Despite recent advances in diagnosis and treatment, iHES remains a clinical challenge, in large part because of its heterogeneous presentation [5,6,7]. Multiple organs may be affected, and patients frequently report nonspecific but disabling symptoms—fatigue, malaise and low-grade fever—that are easily overlooked or misattributed in specialist settings [8].

Owing to the rarity of the disease, only a few studies have reported clinical manifestations in large patient cohorts [9,10,11,12]. The recent literature points to the skin and lung as the most frequently involved organs; cardiac and neurologic complications are less common but well documented. Constitutional symptoms such as fatigue and fever may dominate the clinical picture but are inconsistently classified in observational studies, in part because they are rarely captured with validated patient-reported instruments. Detailed characterisation of organ involvement in longitudinal cohorts is therefore limited, particularly for subtle neurologic manifestations such as cognitive dysfunction or profound fatigue. Moreover, the immunologic underpinnings explaining why some patients develop disease in one organ (e.g., skin) versus another (e.g., heart) are still under investigation, and large-scale data are needed to support the endotyping of iHES.

HES classification has been refined by recent advances in molecular diagnostics. The 2024 WHO/ICC consensus emphasises a molecular framework, prioritising the detection of tyrosine-kinase fusions (FIP1L1-PDGFRA, PDGFRB, FGFR1) and clonal markers, while retaining clinical subtypes such as lymphocytic, idiopathic, and myeloid HES [2]. This molecular stratification has therapeutic implications: fusion-positive disease responds to tyrosine-kinase inhibitors, whereas idiopathic variants may benefit from IL-5 pathway inhibition [13].

Despite their clinical impact, constitutional symptoms such as fatigue, cognitive dysfunction, and mood changes remain poorly characterised in the HES literature. Recent European real-world data suggest that fatigue affects approximately half of patients [14], yet systematic assessment tools are lacking. Better characterisation of these symptoms could improve disease monitoring and quality-of-life evaluation.

The aim of this study was to characterise the clinical picture of iHES at our tertiary referral centre in Poland, with detailed attention to all potential organ involvements previously described in observational studies and case reports. Using an unusually large single-centre cohort accrued over more than eight years, we additionally review the immunologic and molecular mechanisms most plausibly driving the organ-selective patterns observed.

## 2. Materials and Methods

### 2.1. Study Design and Patients

We conducted an analysis of a prospectively maintained single-centre registry of consecutive patients with iHES followed between January 2017 and May 2025 in the Clinical Department of Internal Medicine, Asthma and Allergy of the Medical University of Lodz. During the study period, 198 patients were evaluated for persistent hypereosinophilia (absolute eosinophil count ≥1.5 × 10^9^/L for ≥1 month); after the exclusion of secondary causes and myeloid HES variants, 72 (36%) were classified as iHES and constitute the present cohort. All patients fulfilled the contemporary WHO (2022) and ICC (2024) consensus criteria for HES [2]. Patients with iHES were included after extensive evaluation to exclude secondary causes (parasitic infection, allergic disease, drug reactions, autoimmune and neoplastic disorders) and well-defined HES variants. The systematic workup to exclude secondary eosinophilia comprised a detailed drug and travel history; stool ova-and-parasite examination and relevant parasitic serologies; total and allergen-specific IgE testing; autoimmune and vasculitis serologies (including ANCA); serum tryptase and vitamin B12; peripheral-blood smear review; and cross-sectional imaging. Bone-marrow examination (morphology and cytogenetics) was performed in 41 (57%) patients, after the consulting haematologist deemed it necessary. FIP1L1-PDGFRA fusion was tested by RT-PCR and was negative in all cases, and no patient met the criteria for the lymphocytic variant of HES on flow cytometric assessment for aberrant T-cell immunophenotype. T-cell receptor (TCR) clonality assays and comprehensive myeloid gene panels were not performed; this is acknowledged as a limitation. Echocardiography was systematically performed at baseline in all patients regardless of cardiac symptoms, to ensure the early detection of cardiac involvement.

### 2.2. Data Collection

Demographic and clinical data were extracted from the institutional HES registry, which recorded information collected during routine clinical care. At every clinic visit, each patient was assessed using a structured clinician-administered organ-system checklist developed in 2019 based on prior published case series. The checklist covers eight organ systems (pulmonary, sinonasal, musculoskeletal, gastrointestinal, dermatological, cardiac, neurologic, splenic) and a constitutional-symptom domain (fatigue, low-grade fever, night sweats, weight change, malaise). As the list was developed in 2019, organ-system and constitutional-symptom data for patients first evaluated between 2017 and 2019 were obtained by a structured retrospective review of their medical record. Organ involvement required either clinically apparent disease attributable to eosinophilic inflammation or supportive imaging, endoscopic, or histologic findings. Specifically, pulmonary involvement required eosinophil-attributable lower-airway or parenchymal disease documented by symptoms together with supportive imaging, pulmonary-function, or bronchoscopic/cytologic findings; asthma alone, in the absence of other eosinophil-attributable pulmonary findings, was not considered as pulmonary involvement. Sinonasal involvement required chronic rhinosinusitis with or without nasal polyposis confirmed by an otorhinolaryngologist. Gastrointestinal involvement required eosinophil-attributable symptoms with supportive histologic confirmation. Musculoskeletal involvement was defined as eosinophil-attributable myalgia, arthralgia, arthritis, or myositis after exclusion of alternative causes. Neurologic involvement required objective central or peripheral nervous-system disease (e.g., peripheral neuropathy confirmed on examination/electrophysiology, cerebrovascular events, or encephalopathy) attributable to HES. Cardiac involvement required echocardiographic, biomarker, cardiac-MRI, or histologic evidence of eosinophilic myocardial, endomyocardial, or valvular disease. Dermatological and splenic involvement were defined as eosinophil-attributable skin lesions and imaging-confirmed splenomegaly, respectively. HES flare was defined as a worsening HES-related clinical manifestations and/or increase in peripheral eosinophil count, requiring the escalation of systemic corticosteroids, the addition or escalation of cytotoxic/immunosuppressive therapy, or hospitalisation. The mean number of flares per patient–year reported in Table 1 was derived using this definition. Constitutional symptoms were captured by clinician interview without a validated patient-reported instrument; this is a limitation discussed below. Patient demographics and laboratory features are recorded as summarised in Table 1.

### 2.3. Statistical Analysis

Descriptive statistics were used to summarise the cohort. Continuous variables were presented as the median (IQR) when distributions were skewed and as the mean (SD) when approximately symmetric. Proportions are reported with Wilson 95% confidence intervals. The follow-up duration was calculated from the date of diagnosis to the last recorded visit. The association between the baseline absolute eosinophil count and the number of organ systems involved was assessed by Spearman correlation. The cohort size was insufficient for inferential testing or cluster analyses aimed at endotyping. Statistical analyses were performed using TIBCO Statistica, version 13.3 (TIBCO Software Inc., Palo Alto, CA, USA).

## 3. Results

### 3.1. Patient Characteristics

Of 198 patients evaluated for persistent hypereosinophilia during the study period, 72 (36%) fulfilled the criteria for iHES and were included in the analysis. The median duration of follow-up from diagnosis to the last recorded visit was 46.1 (IQR 38.1–68.2) months. The median age at diagnosis was 45 years (IQR 31–58, range 19–72), and 83% were female. Maximum historical blood eosinophil counts ranged from 1.6 to 12.0 × 10^9^/L (median 4.2 × 10^9^/L). All patients underwent an exhaustive workup for secondary causes. Common causes of reactive eosinophilia—parasitic infection, drug hypersensitivity, and severe atopic disease—were excluded, supporting the idiopathic nature of the cohort. No patient tested positive for FIP1L1-PDGFRA fusion, and bone-marrow cytogenetics revealed no clonal abnormality in any tested case. Serum IgE was elevated (>100 IU/mL) in 40% of patients and serum vitamin B12 was elevated in 25%, while tryptase levels were within normal limits except in one patient who, despite high serum tryptase, had no myeloid mutation identified on further testing.

### 3.2. Organ Involvement Spectrum

**Figure 1:**
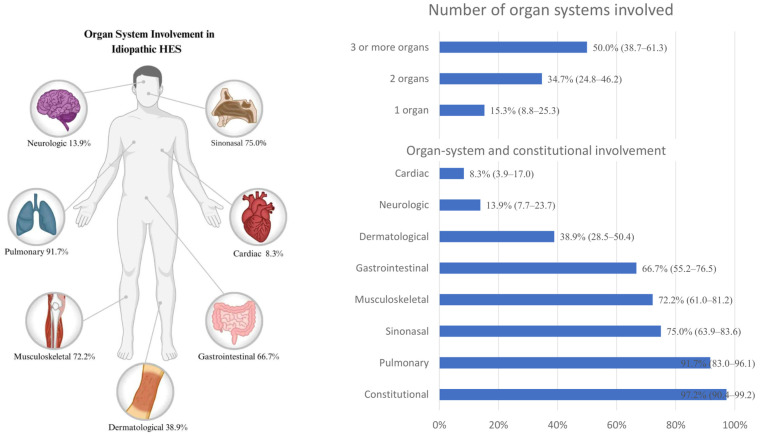
Organ-system involvement frequency among 72 patients with idiopathic hypereosinophilic syndrome (iHES). Left panel: schematic representation of involved organs with corresponding prevalence. Right panel: bar chart showing the proportion of patients with involvement of each organ system and the distribution of the number of organs involved per patient. Percentages with 95% confidence intervals are reported in the main text.

Multi-organ involvement was the rule: the median number of involved organ systems per patient was 3 (IQR 2–4, range 1–6). Single-organ disease occurred in 11 patients (15.3%, 95% CI 8.8–25.3), two-organ involvement in 25 (34.7%, 95% CI 24.8–46.2), and three or more organ system involvement in 36 (50.0%, 95% CI 38.7–61.3). Figure 1 summarises the frequency of involvement by organ system in our cohort.

The most frequently affected organ system was pulmonary (91.7%, 95% CI 83.0–96.1), followed by sinonasal (75.0%, 95% CI 63.9–83.6), musculoskeletal (72.2%, 95% CI 61.0–81.2) and gastrointestinal (66.7%, 95% CI 55.2–76.5). The high pulmonary rate is influenced by referral bias, as our department is a regional severe-asthma reference centre and 80.6% of the cohort had asthma at baseline. Dermatological involvement was observed in 38.9% (95% CI 28.5–50.4), while neurologic and cardiac involvement were less common, affecting 13.9% (95% CI 7.7–23.7) and 8.3% (95% CI 3.9–17.0) of patients, respectively. Constitutional symptoms—most often described as overwhelming fatigue interfering with daily life, together with general malaise—were recorded by clinician interview in 97% of patients (95% CI 90.4–99.2). In exploratory analysis, a higher baseline absolute eosinophil count was not associated with a greater number of involved organ systems (Spearman ρ = −0.035, *p* = 0.84) or with cardiac involvement.

Cardiac involvement, although infrequent, carried clinically significant manifestations, including heart failure with reduced ejection fraction due to eosinophilic myocarditis, apical thrombus formation, and valvular dysfunction from endomyocardial fibrosis. All cardiac cases required aggressive therapy before HES diagnosis (corticosteroids plus anticoagulation or other interventions).

Notable atypical presentations included one patient with extreme hypereosinophilia (33 × 10^9^/L) and isolated cutaneous involvement with dry, flaky skin and purple discoloration; one patient with central-nervous-system involvement presenting as metastasis-like lesions in the brain and spinal cord; and one patient with isolated sinonasal involvement and nasal polyps unresponsive to both systemic and topical glucocorticoid therapy. Imaging and histopathological details for the central-nervous-system case are presented separately as a case report.

## 4. Discussion

Our single-centre cohort confirms that HES is a clinically heterogeneous syndrome and challenges several elements of the “typical” pattern of symptoms and organ involvement reported in earlier observational studies [2,10,11,14,15,16].

The vast majority of our patients had multi-organ involvement, with a median of three organs affected, broadly aligned with the largest contemporary HES studies. The most recent large-scale observation—a five-country European retrospective study—reported a median of three-organ systems involved, with constitutional symptoms (63% of patients), lung involvement (49%), and skin involvement (48%) being the most common manifestations [14].

The most clinically notable finding of our observation was the higher recorded frequency of constitutional and neurologic symptoms compared with historical reports. Prior analyses often under-reported subjective “soft” symptoms such as fatigue [8]. The 188-patient multicentre retrospective study by Ogbogu et al. provided a comprehensive clinical snapshot of HES in which constitutional symptoms—including fatigue, fever, and weight loss—were relatively under-emphasised compared with organ-based manifestations such as cutaneous, pulmonary, or cardiac involvement [11]. By contrast, when fatigue was systematically queried at every visit using a structured checklist, virtually all our patients (97%) reported it during periods of disease activity. This finding most plausibly reflects ascertainment differences rather than a true biological excess: prior series relied largely on volunteered complaints, whereas our protocol actively probed for the symptom. We did not employ a validated patient-reported fatigue instrument (e.g., FSS, FACIT-Fatigue), which limits direct quantitative comparison with cohorts using such tools, and we cannot exclude over-attribution of nonspecific fatigue to HES in some patients. Within these caveats, constitutional symptoms—principally fatigue and flu-like manifestations—appeared, in our clinical experience, to precede measurable rises in eosinophil counts in many patients, although this temporal relationship was not formally analysed.

Active probing for such “silent” manifestations—including screening for neuropathy or cognitive dysfunction—could enable more comprehensive care, and likely accounts for the higher neurologic-involvement rate in our cohort (13.9%) than the ~4–12% range reported in earlier studies [11,17]. Our data reinforce that hypereosinophilia is asymptomatic or “benign” less commonly than previously thought; even when overt organ damage is limited, patients frequently have substantial symptomatic burden not immediately attributed to eosinophilic inflammation. This point is underscored by the review of Requena et al., which highlighted that idiopathic HES patients in the literature often had non-life-threatening but impactful issues such as fatigue that were systematically under-reported [8].

The pattern of organ involvement in our cohort showed both similarities and differences relative to prior studies. Consistent with other series, we found the lungs to be among the most frequently affected organ, while skin was only the fifth-most commonly affected system [18,19]. In the largest aggregate analyses to date, the skin, lung, and gastrointestinal tract emerged as the top three organ systems involved across HES variants [10]. Our data differ from those reports, with pulmonary, sinonasal, and gastrointestinal involvement being the most common manifestations. This pattern almost certainly reflects a referral bias: our department is a regional reference centre for pulmonary diseases and a major severe-asthma treatment facility, and 80.6% of the cohort had asthma at baseline. Earlier studies have noted that skin lesions (rash, angioedema) are particularly prevalent in the lymphocytic variant of HES, which we excluded by aberrant T-cell flow cytometric phenotyping; however, TCR clonality was not assessed, and a fraction of our cohort may have harboured occult clonal T-cell populations that surface immunophenotyping did not capture. The female predominance (83%) is consistent with both severe-asthma referral patterns and with under-detection of lymphocytic-variant disease, both of which warrant explicit acknowledgement [12,20].

Fewer than 10% of our patients had cardiac manifestations at diagnosis, suggesting that cardiac complications often developed later or only in certain subsets (e.g., myeloproliferative forms) [21]. The relatively lower cardiac rate in our series may reflect our focus on idiopathic disease (excluding FIP1L1-PDGFRA-positive cases, which are strongly associated with eosinophilic cardiomyopathy) and possibly the variable follow-up duration. Although cardiac involvement was infrequent (8.3%), its clinical implications were disproportionate to its prevalence: affected patients presented with severe complications, including heart failure due to eosinophilic myocarditis, intracardiac thrombus, and valvular dysfunction from endomyocardial fibrosis. These findings reinforce the well-established concept that cardiac involvement in HES represents one of the most serious and potentially life-threatening manifestations. Importantly, all affected patients required intensive treatment before HES diagnosis, highlighting the risk of delayed recognition. Our observations support the need for systematic cardiovascular screening in all patients with iHES, even in the absence of overt cardiac symptoms. Routine echocardiography and heightened clinical vigilance may permit the earlier detection of subclinical disease and prevention of irreversible cardiac damage. Splenic involvement was also rare, consistent with its association with myeloproliferative variants.

As shown recently by Lefèvre et al., no single-organ involvement is pathognomonic for a particular HES subtype, and iHES itself probably encompasses multiple sub-phenotypes [10]. Our real-world data support this concept of heterogeneity. Even within defined “idiopathic” HES, the spectrum of disease expression ranged from isolated skin involvement to predominantly pulmonary disease and diffuse multi-organ patterns. This variability calls for a deeper investigation of the biological drivers of organ-selective eosinophilic inflammation.

### 4.1. Mechanistic Context

The diverse organ-involvement pattern observed in iHES reflects, at least in part, the varied downstream effects of eosinophil activation in different tissues, although our study did not include cytokine, granule-protein, or molecular interrogation that would allow mechanistic inferences in individual patients. Several plausible contributors are worth noting briefly to frame the heterogeneity our data demonstrate [22,23,24].

First, IL-5 is the dominant cytokine driving eosinophil growth, survival, and tissue recruitment, and IL-5–rich microenvironments are likely a substantial driver of disease in cytokine-driven subsets of iHES [25]. The clinical efficacy of anti-IL-5 biologics in HES, including the steroid-sparing benefit demonstrated in randomised trials of mepolizumab, supports a key pathogenic role for this axis in at least a subset of patients [26]. Second, activated eosinophils release toxic granule contents—major basic protein (MBP), eosinophil cationic protein, and eosinophil peroxidase—that directly damage cells and activate downstream fibrotic and thrombotic pathways [23,27]. This mechanism is best characterised in the heart and endomyocardium, where eosinophil degranulation produces necrosis followed by TGF-β-mediated remodelling and endomyocardial fibrosis [28], and in peripheral nerves, where MBP toxicity contributes to the well-described neuropathic spectrum of HES [29,30]. One of our patients presented with mononeuritis multiplex as the initial manifestation triggering HES workup, consistent with this paradigm.

Third, the molecular and genetic underpinnings of iHES are heterogeneous [31,32]. By definition, our patients had no known primary driver mutation and no clonal lymphocyte phenotype on routine flow cytometry, but TCR clonality and broad myeloid gene panels were not performed. Advances in next-generation sequencing have identified rare myeloid mutations (in genes such as TET2, DNMT3A) in a subset of patients previously labelled idiopathic, blurring the line between truly idiopathic and myeloproliferative HES [33]. Elevated vitamin B12 in 25% of our patients and one case of high serum tryptase suggest that some may harbour latent myeloid neoplasms not captured by our diagnostic workup. As Lefèvre et al. proposed, iHES is best conceived as a protean condition encompassing multiple pathophysiological processes [10], and recognising this complexity is essential for moving toward more personalised management [34,35,36].

### 4.2. Limitations

Several limitations apply to our findings. First, the single-centre design and our institutional role as a regional severe-asthma referral centre introduce a selection bias that almost certainly inflates the observed pulmonary involvement and contributes to the female predominance. Accordingly, the high frequencies of pulmonary, sinonasal, and constitutional involvement may substantially reflect referral patterns rather than the true phenotype of iHES; the observed phenotype may not be representative of the broader iHES population, and comparisons with multicentre cohorts should therefore be interpreted with caution. Second, despite excluding the lymphocytic variant by aberrant T-cell flow cytometric phenotyping, we did not perform TCR clonality assays; some patients may therefore harbour occult clonal T-cell populations not captured by surface immunophenotyping. Similarly, comprehensive myeloid gene panels (e.g., TET2, DNMT3A, ASXL1) were not performed, and a fraction of cases labelled idiopathic may carry occult somatic mutations [37]. Third, fatigue and other constitutional symptoms were captured by structured clinician interviews without a validated patient-reported instrument, preventing direct quantitative comparison with cohorts using such tools and leaving open the possibility of over-attribution. Fourth, cytokine profiling (IL-5, IL-13, TGF-β) was not performed, so any mechanistic interpretation of organ-tropism patterns was necessarily indirect. Fifth, while 72 patients is a respectable single-centre cohort, the statistical power was insufficient for cluster-based endotyping. Sixth, although the cohort was followed longitudinally, most analyses presented here were cross-sectional; the evolution of organ involvement over time, treatment exposure, flare trajectories, and the emergence of new organ manifestations were not systematically analysed and warrant dedicated longitudinal study. Despite these limitations, the rigorous diagnostic criteria, prolonged observation period (>8 years), systematic baseline echocardiography, and structured symptom ascertainment provide a clinically informative description of idiopathic HES.

## 5. Conclusions

In a single-centre cohort of 72 patients with idiopathic HES, a systematic symptom assessment revealed a substantial constitutional-symptom burden—principally fatigue—in nearly all patients, alongside pulmonary, sinonasal, musculoskeletal, and gastrointestinal involvement that were recorded more frequently than in earlier mixed-variant cohorts, although referral-based selection and ascertainment differences limited generalisability. Cardiac involvement was infrequent but uniformly severe, supporting routine baseline echocardiographic screening regardless of cardiac symptoms. The differences between our pulmonary-dominant pattern and skin-dominant patterns in earlier mixed-variant series likely reflect both the idiopathic case definition and selection bias from our severe-asthma referral pattern, illustrating the importance of considering the HES variant in clinical interpretation. Future progress will require validated patient-reported instruments for fatigue and cognitive symptoms, broader genetic and immunological phenotyping (TCR clonality, myeloid gene panels, cytokine profiles), and multicentre cohorts large enough for cluster-based endotyping. The integration of these tools into routine HES care should move the field from descriptive heterogeneity toward mechanism-based subtyping and, ultimately, individualised therapy.

## Figures and Tables

**Table 1:** Baseline demographic and clinical characteristics of the study population (n = 72).

Variable	Value
Age at diagnosis (years), median (IQR)	45 (31–58)
Age at diagnosis (years), mean (SD)	43.8 (18.6)
Time to diagnosis (months), mean (SD)	80.5 (95.2)
Female sex, n (%)	60 (83.3)
Maximum AEC (cells/μL), median (IQR)	4200 (2500–6000)
Yearly HES flares, mean (SD)	2.2 (0.8)
Asthma, n (%)	58 (80.6)
Atopy/allergic disease, n (%)	30 (41.7)
Elevated serum IgE > 100 IU/mL, n (%)	29 (40.3)
Elevated serum vitamin B12, n (%)	18 (25.0)
Baseline Serum vitamin B12 (pg/mL), median (range)	256 (99–758)
Baseline White blood cells (×10^9^/L), median (range)	9.64 (5.00–16.47)
Baseline Haemoglobin (g/L), median (range)	138 (87–165)
Baseline Haematocrit (L/L), median (range)	0.41 (0.27–0.50)
Baseline Red blood cells (×10^12^/L), median (range)	4.60 (3.20–5.70)
Baseline MCV (fL), median (range)	90 (80–104)
Baseline MCH (pg), median (range)	31 (26–35)
Baseline MCHC (g/L), median (range)	336 (316–364)
Baseline Platelets (×10^9^/L), median (range)	271 (170–429)
Baseline Neutrophils, absolute (×10^9^/L), median (range)	3.99 (1.01–8.85)
Baseline Lymphocytes, absolute (×10^9^/L), median (range)	1.96 (0.28–3.96)
Baseline Monocytes, absolute (×10^9^/L), median (range)	0.38 (0.19–0.93)
Baseline Eosinophils, absolute (×10^9^/L), median (range)	1.78 (0.97–4.44)
Baseline Basophils, absolute (×10^9^/L), median (range)	0.06 (0.00–0.24)

AEC, absolute eosinophil count; HES, hypereosinophilic syndrome; IQR, interquartile range; SD, standard deviation. maximum AEC re-expressed as median (IQR) given right-skewed distribution.

## Data Availability

The data presented in this study are available on reasonable request from the corresponding author. The data are not publicly available because of patient privacy considerations.

## Abbreviations

The following abbreviations are used in this manuscript:

AEC	Absolute eosinophil count
ASXL1	Additional sex combs-like 1
CI	Confidence interval
DNMT3A	DNA methyltransferase 3 alpha
FACIT-Fatigue	Functional Assessment of Chronic Illness Therapy–Fatigue
FGFR1	Fibroblast growth factor receptor 1
FIP1L1-PDGFRA	FIP1-like 1–platelet-derived growth factor receptor alpha fusion
FSS	Fatigue Severity Scale
HES	Hypereosinophilic syndrome
IgE	Immunoglobulin E
iHES	Idiopathic hypereosinophilic syndrome
IL-5	Interleukin-5
IL-13	Interleukin-13
IQR	Interquartile range
MBP	Major basic protein
PDGFRB	Platelet-derived growth factor receptor beta
RT-PCR	Reverse transcription polymerase chain reaction
SD	Standard deviation
TCR	T-cell receptor
TET2	Tet methylcytosine dioxygenase 2
TGF-β	Transforming growth factor beta
WHO	World Health Organization

## References

[1] Valent P., Klion A.D., Roufosse F., Simon D., Metzgeroth G., Leiferman K.M., Schwaab J., Butterfield J.H., Sperr W.R., Sotlar K. (2023). Proposed Refined Diagnostic Criteria and Classification of Eosinophil Disorders and Related Syndromes. Allergy.

[2] Shomali W., Gotlib J. (2024). World Health Organization and International Consensus Classification of Eosinophilic Disorders: 2024 Update on Diagnosis, Risk Stratification, and Management. Am. J. Hematol..

[3] Wilkins H.J., Crane M.M., Copeland K., Williams W.V. (2005). Hypereosinophilic Syndrome: An Update. Am. J. Hematol..

[4] Groh M., Rohmer J., Etienne N., Chahla W.A., Baudet A., Wai A.C.H., Chenivesse C., Rusek I.C., Cottin V., Decamp M. (2023). French Guidelines for the Etiological Workup of Eosinophilia and the Management of Hypereosinophilic Syndromes. Orphanet J. Rare Dis..

[5] Kuang F.L., Khoury P., Weller P.F., Wechsler M.E., Klion A.D. (2023). Biologics and Hypereosinophilic Syndromes: Knowledge Gaps and Controversies. J. Allergy Clin. Immunol. Pract..

[6] Curtis C., Ogbogu P.U. (2016). Hypereosinophilic Syndrome. Clin. Rev. Allergy Immunol..

[7] Shi Y., Wang C. (2022). What We Have Learned about Lymphocytic Variant Hypereosinophilic Syndrome: A Systematic Literature Review. Clin. Immunol..

[8] Requena G., van den Bosch J., Akuthota P., Kovalszki A., Steinfeld J., Kwon N., Van Dyke M.K. (2022). Clinical Profile and Treatment in Hypereosinophilic Syndrome Variants: A Pragmatic Review. J. Allergy Clin. Immunol. Pract..

[9] Roufosse F. (2009). Hypereosinophilic Syndrome Variants: Diagnostic and Therapeutic Considerations. Haematologica.

[10] Lefèvre G., Bleuse S., Puyade M., Moulis G., Néel A., Abisror N., Baudet A., Bonnotte B., Dion J., Dossier A. (2025). Hypereosinophilia and Hypereosinophilic Syndromes: First Findings from a Nationwide Multicenter Cohort. Allergy.

[11] Ogbogu P.U., Bochner B.S., Butterfield J.H., Gleich G.J., Huss-Marp J., Kahn J.E., Leiferman K.M., Nutman T.B., Pfab F., Ring J. (2009). Hypereosinophilic Syndromes: A Multicenter, Retrospective Analysis of Clinical Characteristics and Response to Therapy. J. Allergy Clin. Immunol..

[12] Hu Z., Wang W., Thakral B., Chen Z., Estrov Z., Bueso-Ramos C.E., Verstovsek S., Medeiros L.J., Wang S.A. (2021). Lymphocytic Variant of Hypereosinophilic Syndrome: A Report of Seven Cases from a Single Institution. Cytom. B Clin. Cytom..

[13] Klion A.D. (2022). Approach to the Patient with Suspected Hypereosinophilic Syndrome. Hematol. Am. Soc. Hematol. Educ. Program.

[14] Hwee J., Huynh L., Du S., Kwon N., Jakes R.W., Alfonso-Cristancho R., Baylis L., Requena G., Khanal A., Rothenberg M.E. (2023). Hypereosinophilic Syndrome in Europe: Retrospective Study of Treatment Patterns, Clinical Manifestations, and Healthcare Resource Utilization. Ann. Allergy Asthma Immunol..

[15] Roufosse F.E., Goldman M., Cogan E. (2007). Hypereosinophilic Syndromes. Orphanet J. Rare Dis..

[16] Wechsler M.E., Hellmich B., Cid M.C., Jayne D., Tian X., Baylis L., Roufosse F. (2023). Unmet Needs and Evidence Gaps in Hypereosinophilic Syndrome and Eosinophilic Granulomatosis with Polyangiitis. J. Allergy Clin. Immunol..

[17] Moore P.M., Harley J.B., Fauci A.S. (1985). Neurologic Dysfunction in the Idiopathic Hypereosinophilic Syndrome. Ann. Intern. Med..

[18] Long C., Scott J.L., Flamm A. (2023). The Dermatologic and Histologic Spectrum of Hypereosinophilic Syndrome. JAAD Case Rep..

[19] Dulohery M.M., Patel R.R., Schneider F., Ryu J.H. (2011). Lung Involvement in Hypereosinophilic Syndromes. Respir. Med..

[20] Wang X.Q., Shopsowitz K., Lofroth J., Wang X., Peterson E., Weng A.P., Chen L.Y.C. (2025). Lymphocytic Variant Hypereosinophilic Syndrome: Case Series from a Tertiary Referral Center in Canada. eJHaem.

[21] Ogbogu P., Rosing D.R., Horne M.K. (2007). Cardiovascular Manifestations of Hypereosinophilic Syndromes. Immunol. Allergy Clin. N. Am..

[22] Caminati M., Carpagnano L.F., Alberti C., Amaddeo F., Bixio R., Caldart F., De Franceschi L., Del Giglio M., Festi G., Friso S. (2024). Idiopathic Hypereosinophilic Syndromes and Rare Dysimmune Conditions Associated with Hyper-Eosinophilia in Practice: An Innovative Multidisciplinary Approach. World Allergy Organ. J..

[23] Rothenberg M.E. (1998). Eosinophilia. N. Engl. J. Med..

[24] Parrillo J.E., Lawley T.J., Frank M.M., Kaplan A.P., Fauci A.S. (1979). Immunologic Reactivity in the Hypereosinophilic Syndrome. J. Allergy Clin. Immunol..

[25] Taurisano G., Ruffi M.C., Canalis S., Costanzo G.A.M.L. (2025). Hypereosinophilia: Clinical and Therapeutic Approach in 2025. Curr. Opin. Allergy Clin. Immunol..

[26] Valent P., Gleich G.J., Reiter A., Roufosse F., Weller P.F., Hellmann A., Metzgeroth G., Leiferman K.M., Arock M., Sotlar K. (2012). Pathogenesis and Classification of Eosinophil Disorders: A Review of Recent Developments in the Field. Expert Rev. Hematol..

[27] Gleich G.J. (2000). Mechanisms of Eosinophil-Associated Inflammation. J. Allergy Clin. Immunol..

[28] Roufosse F., Kahn J.-E., Rothenberg M.E., Wardlaw A.J., Klion A.D., Kirby S.Y., Gilson M.J., Bentley J.H., Bradford E.S., Yancey S.W. (2020). Efficacy and Safety of Mepolizumab in Hypereosinophilic Syndrome: A Phase III, Randomized, Placebo-Controlled Trial. J. Allergy Clin. Immunol..

[29] Shen Z.-J., Malter J.S. (2015). Determinants of Eosinophil Survival and Apoptotic Cell Death. Apoptosis.

[30] Gleich G.J., Leiferman K.M. (2009). The Hypereosinophilic Syndromes: Current Concepts and Treatments. Br. J. Haematol..

[31] Boussir H., Ghalem A., Ismaili N., El Ouafi N. (2017). Eosinophilic Myocarditis and Hypereosinophilic Syndrome. J. Saudi Heart Assoc..

[32] Takeuchi H., Kawamura K., Kawasaki T., Oka N. (2022). Distinct Features of Hypereosinophilic Syndrome with Neuropathy from Eosinophilic Granulomatosis with Polyangiitis. Front. Neurol..

[33] Wichman A., Buchthal F., Pezeshkpour G.H., Fauci A.S. (1985). Peripheral Neuropathy in Hypereosinophilic Syndrome. Neurology.

[34] Ono R., Iwahana T., Kato H., Okada S., Kobayashi Y. (2021). Literature Reviews of Stroke with Hypereosinophilic Syndrome. Int. J. Cardiol. Heart Vasc..

[35] Stella S., Massimino M., Manzella L., Pennisi M.S., Tirrò E., Romano C., Vitale S.R., Puma A., Tomarchio C., Di Gregorio S. (2021). Molecular Pathogenesis and Treatment Perspectives for Hypereosinophilia and Hypereosinophilic Syndromes. Int. J. Mol. Sci..

[36] Valent P., Horny H.-P., Bochner B.S., Haferlach T., Reiter A. (2012). Controversies and Open Questions in the Definitions and Classification of the Hypereosinophilic Syndromes and Eosinophilic Leukemias. Semin. Hematol..

[37] Lee J.-S., Seo H., Im K., Park S.N., Kim S.-M., Lee E.K., Kim J.-A., Lee J.-H., Kwon S., Kim M. (2017). Idiopathic Hypereosinophilia Is Clonal Disorder? Clonality Identified by Targeted Sequencing. PLoS ONE.

